# Inhibition of PCSK9: A Promising Enhancer for Anti-PD-1/PD-L1 Immunotherapy

**DOI:** 10.34133/research.0488

**Published:** 2024-09-25

**Authors:** Shengbo Sun, Jingxin Ma, Tingting Zuo, Jinyao Shi, Liting Sun, Cong Meng, Wenlong Shu, Zhengyang Yang, Hongwei Yao, Zhongtao Zhang

**Affiliations:** ^1^ Department of General Surgery, Beijing Friendship Hospital, Capital Medical University, State Key Lab of Digestive Health, National Clinical Research Center for Digestive Diseases, Beijing, China.; ^2^ Department of Clinical Laboratory, Beijing Friendship Hospital, Capital Medical University, Beijing, China.; ^3^ College of Biological Sciences and Technology, Yili Normal University, Yining, China.

## Abstract

Immune checkpoint therapy, such as programmed cell death protein 1/programmed death-ligand 1 (PD-1/PD-L1) blockade, has achieved remarkable results in treating various tumors. However, most cancer patients show a low response rate to PD-1/PD-L1 blockade, especially those with microsatellite stable/mismatch repair-proficient colorectal cancer subtypes, which indicates an urgent need for new approaches to augment the efficacy of PD-1/PD-L1 blockade. Cholesterol metabolism, which involves generating multifunctional metabolites and essential membrane components, is also instrumental in tumor development. In recent years, inhibiting proprotein convertase subtilisin/kexin type 9 (PCSK9), a serine proteinase that regulates cholesterol metabolism, has been demonstrated to be a method enhancing the antitumor effect of PD-1/PD-L1 blockade to some extent. Mechanistically, PCSK9 inhibition can maintain the recycling of major histocompatibility protein class I, promote low-density lipoprotein receptor-mediated T-cell receptor recycling and signaling, and modulate the tumor microenvironment (TME) by affecting the infiltration and exclusion of immune cells. These mechanisms increase the quantity and enhance the antineoplastic effect of cytotoxic T lymphocyte, the main functional immune cells involved in anti-PD-1/PD-L1 immunotherapy, in the TME. Therefore, combining PCSK9 inhibition therapy with anti-PD-1/PD-L1 immunotherapy may provide a novel option for improving antitumor effects and may constitute a promising research direction. This review concentrates on the relationship between PCSK9 and cholesterol metabolism, systematically discusses how PCSK9 inhibition potentiates PD-1/PD-L1 blockade for cancer treatment, and highlights the research directions in this field.

## Introduction

Since cancer immunotherapy is discovered through inhibition of immune checkpoints by James P. Allison and Tasuku Honjo [1–3], programmed cell death protein 1 (PD-1) and its ligand (PD-L1) have been considered important “brake” molecules in tumor immunity [4]. When PD-L1 on tumor cells binds to PD-1 on T cells, T cells enter an “exhaustion” state [5,6], and their activities, such as infiltration, activation, and cytokine production, are inhibited [7]. Based on this mechanism, immune checkpoint therapy (ICT) combined with PD-1/PD-L1 blockade prevents PD-1 and PD-L1 from interacting, restoring T cells from an exhausted state and reinvigorating the T-cell-mediated immune response against cancer cells [6]. The PD-1/PD-L1 blockade therapy has shown promising results, such as improved clinical outcomes in a variety of tumors and prolonged survival of treated individuals [8,9]. However, PD-1/PD-L1 inhibitors show substantial antitumor effects in only a small subset of patients, with most individuals exhibiting a low response rate to these treatments [10], especially those with microsatellite stable (MSS)/mismatch repair-proficient (pMMR) colorectal cancer (CRC) subtypes [11]. Consequently, further investigations and more approaches for enhancing the antitumor effect of anti-PD-1/PD-L1 immunotherapy are urgently needed.

**Fig. 1.**
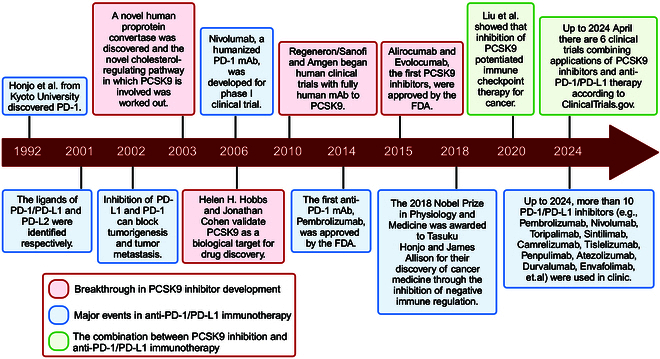
Milestone events of PCSK9 inhibitor and anti-PD-1/PD-L1 immunotherapy. The key findings on PCSK9 inhibitor, the major achievements of anti-PD-1/PD-L1 immunotherapy, and their combination were reviewed retrospectively. FDA, Food and Drug Administration; mAb, monoclonal antibody; PCSK9, proprotein convertase subtilisin/kexin type 9; PD-1, programmed cell death protein 1; PD-L1, programmed death-ligand 1; PD-L2, programmed death-ligand 2. Created with BioRender.com.

Apart from anti-PD-1/PD-L1 immunotherapy, there are close relationships between serum cholesterol levels and tumor development [12]. Cholesterol metabolism, a metabolic process widely present in mammalian cells, involves essential membrane component production and metabolites with various physiological functions [13]. Mechanistically, tumor cells are highly proliferative and require large amounts of nutrients such as cholesterol to meet their proliferative needs [14,15]. In addition, the derivatives and synthesis intermediates of cholesterol are tumor progression regulators [15,16]. As a result, serum cholesterol levels can also affect tumorigenesis to a certain degree. Drugs aimed at cholesterol metabolism for cancer treatment are a rapidly emerging field. In recent years, proprotein convertase subtilisin/kexin type 9 (PCSK9), a serine proteinase that regulates cholesterol metabolism, has shown promising antitumor potential [17]. PCSK9 inhibition may lower serum cholesterol levels, which suggests cholesterol depletion-mediated antitumor effects [17,18]. However, inhibiting PCSK9 has demonstrated enhancing the antitumor effect of PD1/PD-L1 blockade by facilitating the infiltration of cytotoxic T lymphocyte (CTL) and excluding regulatory T cell (Treg) [19]. In 2022, Guo et al. [20] designed a tumor-targeting nanocomposite combining anti-PD1 and anti-PCSK9 effects that have shown both efficacy and safety in CRC therapy. Combining PCSK9 inhibition with anti-PD-1/PD-L1 immunotherapy represents a promising approach for cancer treatment. The key discoveries about PD-1/PD-L1 and PCSK9 are shown in Fig. 1. In addition, the commonly utilized anti-PD-1/PD-L1 inhibitors and PCSK9 inhibitors are summarized in Table 1 [21–39].

**Table 1.** The available anti-PD-1/PD-L1 inhibitors and PCSK9 inhibitors

Target	Name	Approval time	Type	Diseases	Ref.
PD-1	Pembrolizumab	September 2014	Monoclonal antibody	Melanoma, NSCLC, head and neck squamous cell carcinoma, Hodgkin’s lymphoma, urothelial carcinoma, microsatellite instability-high or mismatch repair deficient metastatic (MSI-H/dMMR) CRC, gastric cancer, cervical cancer, and triple-negative breast cancer	[21]
	Nivolumab	December 2014	Monoclonal antibody	Advanced melanoma, NSCLC, renal cell carcinoma, Hodgkin’s lymphoma, head and neck squamous cell carcinoma, urothelial carcinoma, MSI-H/dMMR CRC, and hepatocellular carcinoma	[21]
	Cemiplimab	September 2018	Monoclonal antibody	Locally advanced or metastatic cutaneous squamous cell carcinoma, basal cell carcinoma, and NSCLC	[22]
	Sintilimab	December 2018	Monoclonal antibody	Hodgkin’s lymphoma, NSCLC, hepatocellular carcinoma, and esophageal cancer	[23]
	Toripalimab	December 2018	Monoclonal antibody	Melanoma, nasopharyngeal carcinoma, and urothelial carcinoma	[24]
	Camrelizumab	May 2019	Monoclonal antibody	Hodgkin lymphoma, B-cell lymphoma, esophageal squamous cell carcinoma, gastric and gastroesophageal junction cancer, hepatocellular carcinoma, nasopharyngeal carcinoma, and NSCLC	[25]
	Tislelizumab	December 2019	Monoclonal antibody	Hodgkin’s lymphoma, urothelial carcinoma, gastric and esophageal cancer, lung cancer, ovarian, fallopian tube or peritoneal cancers, and hepatocellular carcinoma	[26]
	Dostarlimab	April 2021	Monoclonal antibody	MSI-H/dMMR recurrent or advanced endometrial cancer and dMMR advanced solid tumors	[27]
PD-L1	Atezolizumab	May 2016	Monoclonal antibody	Urothelial carcinoma, NSCLC, triple-negative breast cancer, metastatic renal cell carcinoma, and CRC	[28]
	Avelumab	March 2017	Monoclonal antibody	Merkel cell carcinoma, urothelial carcinoma, and renal cell carcinoma	[29]
	Durvalumab	May 2017	Monoclonal antibody	Advanced biliary tract cancer, NSCLC, small cell lung cancer, hepatocellular carcinoma	[30]
	Envafolimab	November 2021	Single-domain antibody	CRC, gastric cancer, and gastric or gastroesophageal junction cancer	[31]
	Adebrelimab	February 2023	Monoclonal antibody	Esophageal squamous cell carcinomas, small cell lung cancer and NSCLC	[32]
PCSK9	Alirocumab	July 2015	Monoclonal antibody	Hyperlipidemia and heterozygous familial hypercholesterolemia	[33]
	Evolocumab	July 2015	Monoclonal antibody	Hyperlipidemia, heterozygous, or homozygous familial hypercholesterolemia	[34]
	Inclisiran	December 2020	siRNA	Primary hypercholesterolemia and mixed dyslipidemia	[35]
	Tafolecimab	August 2023	Monoclonal antibody	Hyperlipidemia and hypercholesterolemia	[36]
	Ongericimab	Not approved	Monoclonal antibody	Primary hypercholesterolemia and mixed dyslipidemia	[37]
	Recaticimab	Not approved	Monoclonal antibody	Hypercholesterolemia	[38]
	Ebronucimab	Not approved	Monoclonal antibody	Primary hypercholesterolemia and mixed dyslipidemia	[39]

CRC, colorectal cancer; MSI-H/dMMR, microsatellite instability-high/mismatch repair-deficient; NSCLC, non-small cell lung cancer.

In the present review, the effects of cholesterol on tumor growth and altered cholesterol metabolism in tumor cells were first described. We then explained the relationship between cholesterol metabolism and PCSK9 inhibition and elucidated the cholesterol depletion-mediated antitumor effects of PCSK9 inhibition. The detailed antitumor mechanisms of PCSK9 inhibition combined with PD-1/PD-L1 blockades are also discussed. This review summarizes the latest developments and prospects of adjunctive therapy with anti-PCSK9 and anti-PD-1 antibodies, aiming to provide novel insights for enhancing the antitumor effect of ICT.

## Mutual Influence of Cholesterol and Cancer

Cholesterol, a crucial element of biological membranes and lipid rafts, is important in tumor proliferation and development. Cholesterol metabolism regulates the physiological processes of tumor cells, especially tumorigenic signaling pathways, ferroptosis, and the tumor microenvironment (TME) [40,41]. In addition to being regulated by cholesterol metabolism, tumor cells can also alter their own cholesterol metabolism. The cholesterol metabolism processes are reprogrammed in a direction that favors tumor growth and development. Therefore, there is a mutual influence between cholesterol and cancer.

### Role of cholesterol in tumor development

**Fig. 2.**
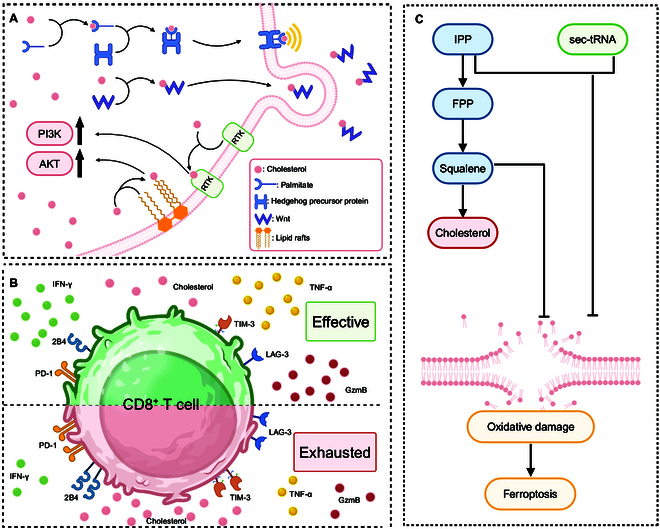
Role of cholesterol in tumor development. (A) Signaling pathways regulated by cholesterol in tumor cells. Cholesterol performs functions in tumor development via Hedgehog, Wnt, and PI3K/AKT signaling pathways. (B) Cholesterol affects the TME. The up-regulated cholesterol in the TME may cause CD8^+^ T-cell exhaustion via up-regulated expression of immune checkpoints, thereby reducing cytokine secretion. (C) Cholesterol and ferroptosis. Cholesterol indirectly affects ferroptosis via the intermediate metabolites and enzymes during cholesterol biosynthesis. FPP, farnesyl pyrophosphate; GzmB, granzyme B; IFN-γ, interferon-γ; IPP, isopentenyl-pyrophosphate; LAG-3, lymphocyte activation gene-3; PD-1, programmed cell death protein 1; PI3K/AKT, phosphoinositide 3-kinase/protein kinase B; RTK, receptor tyrosine kinase; sec-tRNA, selenocysteine-specific transfer ribonucleic acid; TIM-3, T-cell immunoglobulin domain and mucin domain-3; TME, tumor microenvironment; TNF-α, tumor necrosis factor-α; Wnt, Wingless and Int-1. Created with BioRender.com.

In recent years, numerous studies have demonstrated that cholesterol is a mediator that regulates various signaling pathways in tumor cells. The Hedgehog signaling pathway is a key factor in tumor proliferation. The Hedgehog signaling pathway activation can lead to increased proliferation of stem or progenitor cells in tissues derived from the ectoderm [42,43]. In this pathway, cholesterol is a necessary material for the Hedgehog ligand modification [44]. It combines with palmitate to form a dual lipid adduct and attaches to the Hedgehog precursor protein [45]. After modification, the mature Hedgehog protein binds to the cell membrane and performs subsequent functions [42,46]. In addition, cholesterol also functions in the Wingless and Int-1 (Wnt) signaling pathway, which is compatible with Hedgehog signaling and plays an important role in cell proliferation, polarity, and differentiation [47–50]. The phosphoinositide 3-kinase/protein kinase B (PI3K/AKT) pathway is another important signaling pathway involved in regulating cell survival, growth, metabolism, and angiogenesis [51]. Cholesterol can activate the PI3K/AKT pathway by binding to and stabilizing receptor tyrosine kinase (RTK), particularly epidermal growth factor receptor (EGFR) and ErbB2, which are overexpressed in many cancers [52]. Cholesterol can also modulate the localization and activity of AKT by affecting lipid rafts, the cholesterol-rich regions on the cell membrane that serve as platforms for signal transduction [53]. Other signaling pathways activated by increased cholesterol, such as the mitogen-activated protein kinase (MAPK), Kirsten rat sarcoma viral oncogene homolog/mitogen-activated protein kinase/extracellular signal-regulated kinase (KRAS/MEK/ERK), and Janus kinase 2/signal transducer and activator of transcription 3 (JAK2/STAT3) pathways, have also been shown to be related to tumorigenesis [12]. Thus, cholesterol performs indispensable functions in tumor development via various tumorigenic signaling pathways (Fig. 2A).

The TME is a special metabolic unit that includes cellular components (tumor cells, immune cells, and stromal cells) and intercellular contents [54]. Recent studies have increasingly shown that the TME is crucial in tumor occurrence, development, migration, recurrence, and therapeutic efficacy [55]. It is widely accepted that the TME is a key evolutionary and physiological process in tumor progression and cancer therapy and possesses great research and clinical prospects [56]. Cholesterol metabolism sensibly affects immune cell functions in the TME [57]. Cholesterol-derived metabolites facilitate various tumor-associated events in the tumor-infiltrating immune cells (TIICs) [58]. Cholesterol is also reported to cause CD8^+^ T-cell exhaustion in the TME via up-regulated levels of inhibitory receptors such as PD-1, lymphocyte activation gene-3 (LAG-3), T-cell immunoglobulin domain and mucin domain-3 (TIM-3), and 2B4 (CD244), which are affiliated with the expression of cytokines such as interferon-γ (IFN-γ), tumor necrosis factor-α (TNF-α), and granzyme B (GzmB) [59]. In addition, the level of cholesterol in the TME is closely correlated with the low-density lipoprotein receptor (LDLR), a key membrane receptor regulating tumor growth, and the process of cholesterol metabolism is reprogrammed in tumor cells [60,61]. The relevant mechanisms will be systematically discussed in the following sections. Thus, cholesterol also has nonnegligible effects on the TME (Fig. 2B).

Ferroptosis has emerged as a prominent topic in cancer research since it was defined as an iron-dependent regulated cell death (RCD) by Dixon et al. in 2012 [62,63]. In tumor cells, normal iron metabolism is reprogrammed to elevate the level of free iron to satisfy growth needs [64]. Although the function of cholesterol in ferroptosis remains ambiguous, some intermediate metabolites and enzymes involved in the biosynthesis of cholesterol have demonstrated a crucial role [65]. The intermediate metabolites include isopentenyl-pyrophosphate (IPP), farnesyl pyrophosphate (FPP), and squalene. IPP is a component of selenoprotein synthesis and is related to selenocysteine-specific transfer ribonucleic acid (sec-tRNA) stabilization, thereby facilitating ferroptosis inhibition [66,67]. FPP is a precursor of coenzyme Q10 (CoQ10), which sensibly suppresses the ferroptosis [68,69]. Squalene epoxidase (SQLE) is a key enzyme involved in cholesterol biosynthesis. SQLE loss causes squalene accumulation in cholesterol auxotrophic lymphomas, thereby inhibiting ferroptosis [70]. These findings suggest that cholesterol indirectly affects ferroptosis via intermediate metabolites and enzymes during cholesterol biosynthesis (Fig. 2C).

### Altered cholesterol metabolism in cancer

In normal cells, cholesterol metabolism, involving biosynthesis, uptake, efflux, and storage, is a necessary and complicated biochemical process [15,71]. The cholesterol biosynthesis pathway initiates with the mitochondrial production of acetyl-CoA (acetyl coenzyme A). This compound condenses to form acetoacetyl-CoA (acetoacetyl coenzyme A), which is then transformed into HMG-CoA (3-hydroxy-3-methylglutaryl coenzyme-A) by HMG-CoA synthase. In the cytoplasm, HMG-CoA reductase (HMGCR), a rate-limiting enzyme, reduces HMG-CoA to mevalonate (MVA). MVA is phosphorylated and decarboxylated to IPP, which polymerizes into FPP. FPP dimerizes to squalene, which cyclizes to lanosterol, the precursor to cholesterol via Bloch or Kandutsch–Russel pathways [55]. The absorption process of cholesterol uptake is facilitated by Niemann–Pick C1-like1 (NPC1L1) proteins in the intestine and LDLR in the plasma [72,73]. NPC1L1, a glycosylated membrane protein with multiple spans, is uniquely expressed on the apical region of enterocytes and human hepatocytes, while LDLR is expressed on the basolateral surface of most cells. Cholesterol uptake from extracellular sources is mediated by both pathways via the clathrin-dependent mechanism [72]. The clathrin-mediated pathway in mammalian cells is the major endocytic pathway that transforms ligand proteins via clathrin-coated vesicles [74]. Biosynthesis and uptake are the 2 main pathways related to tumor development.

**Fig. 3.**
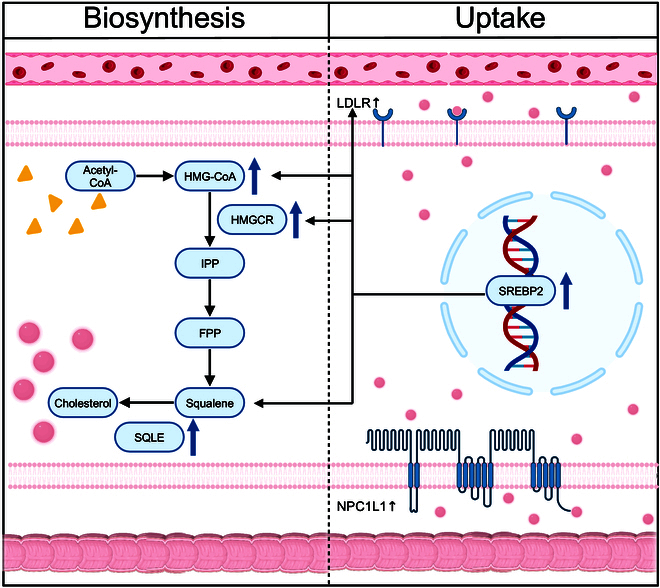
Altered cholesterol metabolism in cancer. Cholesterol biosynthesis and uptake are enhanced in tumor cells. These processes are reprogrammed in a direction that favors tumor growth and development. Acetyl-CoA, acetyl coenzyme A; FPP, farnesyl pyrophosphate; HMG-CoA, 3-hydroxy-3-methylglutaryl coenzyme-A; HMGCR, HMG-CoA reductase; IPP, isopentenyl-pyrophosphate; LDLR, low-density lipoprotein receptor; NPC1L1, Niemann-Pick C1-like1; SQLE, squalene epoxidase; SREBP, sterol regulatory element binding protein. Created with BioRender.com.

In tumor cells, cholesterol metabolism processes are reprogrammed in a direction that favors tumor growth and development. Since cholesterol is beneficial for the growth and development of tumor cells [14,75], tumor cells can increase cholesterol levels in various ways, thereby satisfying their own growth needs. For example, cholesterol biosynthesis and uptake are enhanced in tumor cells [76,77]. Several enzymes regulating cholesterol biosynthesis, such as HMGCR and sterol regulatory element binding protein (SREBP), are substantially elevated in various cancers [78–80]. SREBP includes 3 isoforms, SREBP1a, SREBP1c, and SREBP2 [81]. SREBP1 is a regulator of fatty acid synthesis, and SREBP2 is related to cholesterol biosynthesis initiation and uptake [82]. SQLE is also sensibly up-regulated in pancreatic cancer [83], promoting squalene cyclization in cholesterol biosynthesis. Some signaling pathways involving cholesterol biosynthesis can also be overactivated in tumor cells. The PI3K/AKT pathway is one of these pathways [84], and its activation contributes to tumor occurrence, proliferation, invasion, and migration [51]. However, some tumor cells can accelerate cholesterol uptake via NPC1L1. NPC1L1-knockout mice reportedly acquire resistance to colorectal tumorigenesis [85]. Interestingly, some lymphoma cells acquire cholesterol in a manner dependent on the cholesterol uptake pathway. These cells satisfy their proliferative needs by up-regulating the expression level of LDLR [70], thereby promoting cholesterol uptake via LDLR-mediated absorption in plasma. In addition, tumor cells can maintain high cholesterol levels in the TME by dysregulating cholesterol efflux [86,87] and enhancing cholesterol esterification [88,89]. More details can be found in Refs. [14, 15]. In brief, tumor cholesterol metabolism can be altered in various ways (Fig. 3). Targeting altered cholesterol metabolism represents a promising choice for tumor treatment.

## Inhibition of PCSK9 Suppresses Tumor Growth

**Fig. 4.**
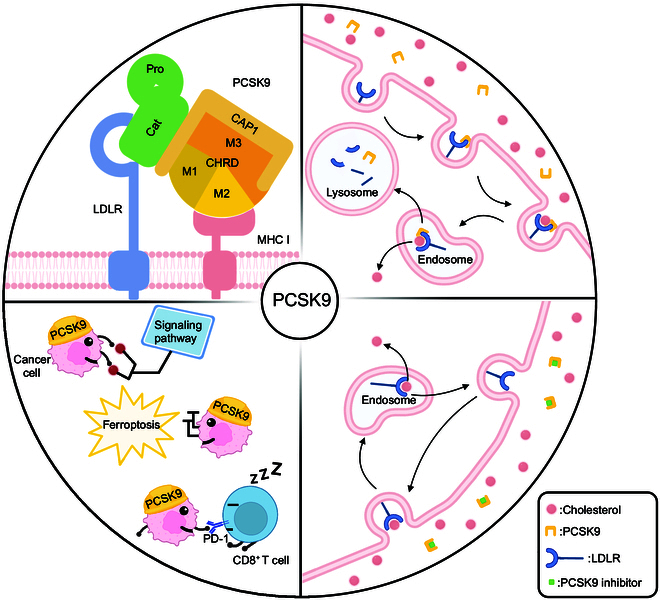
The biology of PCSK9. The biological structure of PCSK9 (upper left). The physiological role of PCSK9 in cholesterol metabolism. LDLR combines with LDL and transports it from plasma to endosome. After releasing LDL in the endosome, LDLR will recycle to the plasma membrane and continue to transport LDL for degradation. PCSK9 may bind to LDLR and directs it to the lysosome for degradation after LDL release. The process can be blocked if a PCSK9 inhibitor combines with PCSK9 (upper right and lower right). PCSK9 promotes tumorigenesis. Corresponding to the effects of cholesterol on tumor growth, PCSK9 may indirectly promote tumorigenesis by up-regulating the tumorigenic signaling pathway, resisting ferroptosis, and affecting the TME (lower left). CAP1, cyclase-associated protein 1; CHRD, C-terminal Cys/His-rich domain; LDLR, low-density lipoprotein receptor; MHC I, major histocompatibility protein class I; PCSK9, proprotein convertase subtilisin/kexin type 9; PD-1, programmed cell death protein 1. Created with BioRender.com.

Proprotein convertases (PCs) are a specialized class of serine proteinases that facilitate the activation of inactive secretory protein precursors [90]. PC cleaves these precursors and converts them into active peptide/protein or multifunctional fragments [90]. PCSK9, which belongs to the PC family, is the ninth member programmed by the gene PCSK9 located on chromosome 1 [91,92]. Since PCSK9 was first reported in 2003 [92], it has become a promising target for treating dyslipidemia [93]. Research indicates that inhibiting PCSK9 reduces serum low-density lipoprotein cholesterol (LDL-c) by promoting the degeneration of LDLR [94,95]. Currently, evolocumab and alirocumab, 2 monoclonal antibodies targeting PCSK9, have received Food and Drug Administration (FDA) approval for treating hypercholesterolemia [96]. Since serum cholesterol levels are closely related to tumor proliferation and development, the inhibition of PCSK9 can suppress tumor growth to a certain extent by lowering serum cholesterol levels (Fig. 4).

### Biological structure of PCSK9

The functions of enzymes are closely related to their biological structure. Determining the biological structure of PCSK9 is essential before understanding its role in cholesterol metabolism. The 3-dimensional structure of PCSK9 includes 3 distinct functional regions: a prodomain, a catalytic domain, and a C-terminal cysteine/histidine-rich domain (CHRD) [97,98]. After secretion from the endoplasmic reticulum (ER), PCSK9 remains connected to its prodomain [92]. The prodomain plays a vital role in the efflux of PCSK9 from the ER [99]. By interacting with LDLR, the catalytic subunit directs it toward lysosomal degradation, which prevents LDLR from circulating to the cell surface [100,101]. The biological function of PCSK9 mainly depends on the catalytic subunit. CHRD consists of 3 tandem repeats, namely, M1, M2, and M3. The M2 repeat interacts with the major histocompatibility protein class I (MHC I) [99]. Besides, cytosolic adenyl cyclase-associated protein 1 (CAP1), a protein with structural homology to CHRD, is considered to bind to the M1 and M3 domains, thereby enhancing PCSK9 activity and LDLR degradation [102,103]. More details of the biological structure of PCSK9 are available in the literature [99].

### Physiological role of PCSK9 in cholesterol metabolism

For decades, PCSK9 has played a vital role in increasing serum cholesterol levels [104]. The physiology of PCSK9 is well developed by researchers, and its mechanisms are widely used for treating atherosclerosis and preventing atherosclerotic cardiovascular events [104]. PCSK9 promotes LDLR degradation mainly via an extracellular mechanism [105,106]. In this pathway, the interaction occurs between PCSK9 secreted into plasma and LDLR located on the plasma membrane. The EGF-like repeat homology domain-A (EGF-A) and β-propeller domain of LDLR interacts with the catalytic subunit of secreted PCSK9 [97,107]. The N-terminal region of the first EGF-A domain of LDLR is specifically bound by the catalytic domain of PCSK9. This interaction occurs at the plasma membrane and requires the presence of calcium ions, resulting in the generation of the PCSK9/LDLR composite [108]. After that, the complex combined with LDL is transferred from the cell membrane to the endosome via a clathrin-mediated pathway. In addition, experiments demonstrated that the PCSK9/LDLR complex interacting with CAP1 can enter cells by caveolin-dependent endocytosis [103]. Within endosomes, the acidic conditions sensibly boost the binding affinity of PCSK9 and LDLR due to electrostatic interactions [109,110]. Subsequently, LDL detaches from LDLR, and PCSK9 targets LDLR to the lysosome for degradation [111]. If PCSK9 does not bind to LDLR on the plasma membrane, after LDL is released into the endosome, LDLR will be recycled to the hepatocyte membrane and continue to deliver LDL for degradation [111]. In addition, some evidence shows that PCSK9 also enhances LDLR degradation via an intracellular pathway [112]. In this pathway, newly synthesized PCSK9 interacts with intracellular LDLR in the trans-Golgi network and directly targets it for the lysosomal degradation [113,114]. Poirier et al. [112] reported that blocking the transport of molecules from the trans-Golgi network to lysosomes via clathrin light-chain siRNAs (small interfering ribonucleic acid) sensibly prevented LDLR degradation in a PCSK9-dependent manner. Therefore, PCSK9 can increase serum cholesterol levels by enhancing the degradation of LDLR via both extracellular and intracellular pathways.

### Inhibition of PCSK9 promotes cholesterol depletion-mediated antitumor effects

Cholesterol is a universal element of the cell membrane and plays a regulatory role in tumorigenesis. In contrast, current studies suggest that higher serum cholesterol levels are associated with an increased risk and extent of tumor progression [12]. A Korean prospective study found that higher serum cholesterol levels are linked to prostate and colon cancer in men, as well as breast cancer in women [115]. Additionally, a Mendelian randomization study showed that higher serum LDL levels are linked to an increased risk of renal cancer [116]. On the other hand, PCSK9 is a PC that facilitates LDLR degradation. Inhibition of PCSK9 promotes LDLR recycling instead of degradation, thereby lowering serum cholesterol levels through the cholesterol uptake process. Thus, the inhibition of PCSK9 can suppress tumor development to a certain degree by lowering serum cholesterol levels. Targeting PCSK9 is not only effective for the treatment of hypercholesterolemia but also promising for cancer therapy.

Corresponding to the effects of cholesterol on tumor growth, PCSK9 inhibition also has cholesterol depletion-mediated antitumor effects by regulating tumorigenic signaling pathways, ferroptosis and the TME. For example, Jin et al. reported that flubendazole, an anthelmintic drug, inhibited the Hedgehog signaling pathway by directly interacting with PCSK9 [117]. Wang et al. [118] demonstrated that PCSK9 facilitates the progression and metastasis of colon cancer by regulating epithelial–mesenchymal transition (EMT) and the PI3K/AKT signaling pathway. Shu et al. [119] showed that Lin28b, an RNA-binding protein, promoted the growth of pancreatic ductal adenocarcinoma by inducing PCSK9’s production. These findings reflected the mutual influence between PCSK9 and tumorigenic signaling pathways. Regarding the role of PCSK9 in ferroptosis, Alannan et al. [120] indicated that PCSK9 inhibition enhanced the vulnerability of liver cancer cells to iron-triggered lipid peroxidation, thereby triggering cell death by ferroptosis. Zhuang et al. [121] demonstrated that in abdominal aortic aneurysm neck, PCSK9 was highly expressed and some ferroptosis-related genes were also down-regulated, indicating the relationship between PCSK9 and the ferroptosis-related genes. Moreover, Yuan et al. [122] reported that PCSK9 inhibits LDLR-mediated T-cell receptor (TCR) recycling and signaling, thus down-regulating CD8^+^ T-cell-mediated antitumor response. The results suggested that PCSK9 also plays a role in the TME. Therefore, PCSK9 inhibition might offer a novel strategy for preventing and treating cancer by modulating cholesterol metabolism. However, further research is required to clarify the underlying mechanisms and clinical implications of PCSK9 in cancer biology [123].

## Inhibition of PCSK9 Potentiates Anti-PD-1/PD-L1 Immunotherapy

**Fig. 5.**
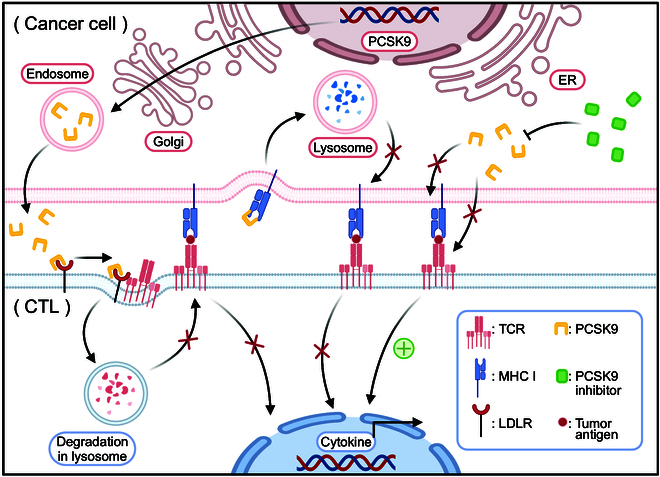
PCSK9 prevents MHC I and LDLR-mediated TCR recycling. PCSK9 binds to MHC I and directs it to the lysosomal pathway for degradation instead of being recycled back to the cell surface. PCSK9 can also interfere with TCR recycling and signaling by binding to LDLR and preventing its interaction with TCR. By blocking the LDLR–TCR interaction, PCSK9 causes TCR to be sorted into the lysosomal pathway instead of being recycled back to the cell surface. The PCSK9 inhibitor may block these processes. CTL, cytotoxic T lymphocyte; ER, endoplasmic reticulum; LDLR, low-density lipoprotein receptor; MHC I, major histocompatibility protein class I; PCSK9, proprotein convertase subtilisin/kexin type 9; TCR, T-cell receptor. Created with BioRender.com.

Apart from restraining tumor growth via the cholesterol depletion-mediated pathway, more studies have shown that PCSK9 inhibition can also potentiate the effects of PD-1/PD-L1 blockades, which suggests a novel approach for tumor treatment (Table 2). Anti-PD-1/PD-L1 immunotherapy is a type of cancer treatment that blocks the interaction between PD-1 and PD-L1 and restores the T-cell antitumor function [124]. However, the effectiveness of this therapy is restricted by the minimal expression of PD-L1 on tumor cells and the immunosuppressive nature of the TME [125]. It has been proven that inhibition of PCSK9 can maintain MHC I recycling and facilitate LDLR-mediated TCR recycling and signaling, thereby improving the effectiveness of PD1/PD-L1 blockades (Fig. 5) [122,126]. Moreover, PCSK9 inhibition can also modulate the TME by affecting the infiltration and exclusion of immune cells [19].

**Table 2.** Studies on enhancing anti-PD-1/PD-L1 immunotherapy via PCSK9 inhibition

Title	Cancer type	Conclusion	Year	Journal	PMID
Inhibition of PCSK9 potentiates immune checkpoint therapy for cancer	Melanoma, colon cancer, and breast cancer	By physically binding to MHC I, PCSK9 interferes with its recycling to the cell surface, leading to its redirection and degradation within the lysosome.	2020	*Nature*	33177715
Potentiating CD8^+^ T cell antitumor activity by inhibiting PCSK9 to promote LDLR-mediated TCR recycling and signaling	CRC, lung cancer, and breast cancer	The TME suppresses the levels of LDLR in CD8^+^ T cells and their TCR signaling through tumor cell-derived PCSK9. Genetic deletion or pharmacological inhibition of PCSK9 in tumor cells appears to improve the antitumor response of CD8^+^ T cells by alleviating their suppression.	2021	*Protein & Cell*	33606190
Self-assembly of a multifunction DNA tetrahedron for effective delivery of aptamer PL1 and Pcsk9 siRNA potentiate immune checkpoint therapy for colorectal cancer	CRC	The multifunctional TDN-FA/PL1/Pcsk9-siRNA demonstrates efficacy and safety in CRC, thereby broadening the utilization of DNA nanotechnology for novel treatments across different cancer types.	2022	*ACS Applied Materials & Interfaces*	35817627
Inhibition of PCSK9 enhances the antitumor effect of PD-1 inhibitor in colorectal cancer by promoting the infiltration of CD8+ T cells and the exclusion of Treg cells	CRC	Neutralizing PCSK9 in conjunction with anti-PD-1 therapy produces a synergistic effect against tumors, characterized by enhanced infiltration of CD8^+^ T cells and inflammatory cytokine secretion. Additionally, the simultaneous inhibition of PCSK9 and PD-1 sensibly reduces the population of Treg cells.	2022	*Frontiers in Immunology*	36003387
PCSK9 regulates the efficacy of immune checkpoint therapy in lung cancer	Lung cancer	High PCSK9 expression in baseline tumor tissue was associated with reduced efficacy of anti-PD-1 immunotherapy in advanced NSCLC patients. The PCSK9 inhibitor combining with the anti-CD137 agonist not only enhanced the recruitment of CD8^+^ T cells but also reduced Tregs.	2023	*Frontiers in Immunology*	37025995
Targeting PCSK9 reduces cancer cell stemness and enhances antitumor immunity in head and neck cancer	Head and neck cancer	PCSK9 levels were found to be elevated in tumor tissues correlating with poorer prognosis in patients. Reducing PCSK9 expression through pharmacological inhibitors or siRNA could inhibit the cancer cells’ stemness-like characteristics in a LDLR-dependent process. Inhibiting PCSK9 increased the infiltration of CD8^+^ T cells and enhanced the efficacy of anti-PD-1 immunotherapy against tumors.	2023	*iScience*	37305703
Fumarate hydratase enhances the therapeutic effect of PD-1 antibody in colorectal cancer by regulating PCSK9	CRC	In mice with low Fh1 expression, the efficacy of PD-1 antibodies was enhanced when the treatment was combined with a PCSK9 inhibitor. CRC patients with low FH expression might experience improved outcomes from a combined therapy of PD-1 antibodies and PCSK9 inhibitors.	2024	*Cancers*	38398104
Multiple immunomodulatory strategies based on targeted regulation of proprotein convertase subtilisin/kexin type 9 and immune homeostasis against hepatocellular carcinoma	Hepatocellular cancer	This research introduces CaCO_3_-based nanoparticles that employ various immunomodulatory strategies against hepatocellular carcinoma. These strategies involve inhibiting PCSK9 and adjusting immune balance within the challenging TME.	2024	*ACS Nano*	38466366

CRC, colorectal cancer; FA, folic acid; FH, fumarate hydratase; LDLR, low-density lipoprotein receptor; MHC I, major histocompatibility protein class I; NSCLC, non-small cell lung cancer; TCR, T-cell receptor; TDN, DNA tetrahedral nanoparticles; TME, tumor microenvironment; Treg, regulatory T cell.

### PCSK9 disrupts the recycling of MHC I by promoting MHC I degradation

MHC I is a membrane-bound glycoprotein crucial in antigen presentation and immune recognition [127]. Intracellular protein-derived peptides are presented to CD8^+^ T cells by MHC I molecules, which can then activate cytotoxic responses against infected or neoplastic cells [127]. The function and expression of MHC I are regulated by diverse factors, including the availability of peptide ligands, the activity of the antigen-processing machinery, and the recycling of MHC I molecules from the endosomal compartment to the cell membrane [128].

PCSK9 influences cholesterol metabolism by attaching to LDLR and directing it to lysosomes for degradation. However, PCSK9 interacts with other membrane receptors, such as MHC I, and affects their trafficking and stability [126]. The molecular mechanism by which PCSK9 disrupts the recycling of MHC I is similar to that of LDLR [126]. PCSK9 binds to the EGF-A domain of MHC I and competes with beta-2-microglobulin (β_2_m) for the same binding site [129]. By physically blocking the MHC I–β_2_m interaction, PCSK9 causes MHC I to be sorted into the lysosomal pathway instead of recycled back to the cell surface [126]. In the lysosome, MHC I is degraded by acid hydrolases, resulting in reduced MHC I expression and impaired antigen presentation [127]. This mechanism is independent of the role of PCSK9 in regulating LDLR levels, as it occurs even in the presence of cholesterol supplementation [126].

The disruption of MHC I recycling by PCSK9 has important implications for cancer immunotherapy, as it reduces the capacity of tumor cells to present antigens to CD8^+^ T cells and evade immune surveillance. By inhibiting PCSK9, either genetically or pharmacologically, MHC I recycling is restored, and antigen presentation is enhanced. This improves the effectiveness of ICT for cancer, such as anti-PD-1/PD-L1 blockade, which aims to revive the antitumor activity of T cells by preventing the PD1 and PD-L1 interaction [126]. PCSK9 inhibition can also synergize with other strategies that increase the expression or function of MHC I, such as IFN-γ, histone deacetylase inhibitors, or proteasome inhibitors [99,126]. Therefore, targeting PCSK9 represents a novel approach to modulate MHC I expression and enhance the antitumor effect of ICT.

### Promotion of LDLR-mediated recycling and signaling

In addition to disrupting the recycling of MHC I, PCSK9 also affects the recycling and signaling of TCR, which are essential for the activation and function of T cells [122]. The TCR is a heterodimeric protein complex comprising α and β chains that recognize peptide–MHC complexes on antigen-presenting cells and initiate intracellular signaling cascades that lead to T-cell proliferation, differentiation, and cytokine production [130]. The expression and function of TCR are regulated by diverse factors, including the availability of peptide ligands, the activity of the antigen-processing machinery, and the recycling of TCR from the endosomal compartment to the cell membrane [131,132].

LDLR mediates the uptake of LDL and regulates cholesterol metabolism. However, LDLR also plays a role in TCR recycling and signaling, as it interacts with TCR and facilitates its retrieval from endosomes to the plasma membrane [122,133]. LDLR is located on T-cell membranes and binds to TCR via its EGF-A domain [134]. This interaction takes place at the plasma membrane and necessitates the presence of calcium ions, resulting in the formation of the LDLR–TCR complex [134,135]. After that, the complex is taken into the endosome via clathrin-mediated endocytosis [122]. In the endosome, LDLR and TCR dissociate, and LDLR returns to the cell surface, transporting TCR [122]. This process allows TCR to be reused for antigen recognition and signaling, thereby enhancing the antitumor activity of T cells.

PCSK9 regulates cholesterol metabolism by attaching to LDLR and directing it to lysosomal degradation. However, PCSK9 can also interfere with TCR recycling and signaling by attaching to LDLR and preventing its interaction with the TCR [122]. PCSK9 interacts with the EGF-A domain of LDLR and competes with TCR for the same binding site [136]. By blocking the LDLR–TCR interaction, PCSK9 causes TCR to be sorted into the lysosomal pathway instead of returning to the cell surface. In the lysosome, TCR is degraded by acid hydrolases, resulting in reduced TCR expression and impaired antigen recognition and signaling [122]. This process is separate from PCSK9’s function in controlling MHC I levels, as it happens even without MHC I or in cells that do not express MHC I [122].

The interference of TCR recycling and signaling by PCSK9 has important implications for cancer immunotherapy, as it reduces the capacity of T cells to respond to tumor antigens and exert cytotoxic effects [137]. By inhibiting PCSK9, either genetically or pharmacologically, TCR recycling and signaling are restored, and the antitumor activity of T cells is potentiated [122]. This improves the effectiveness of ICT for cancer, including PD-1/PD-L1 blockade, which intends to revive the antitumor activity of T cells by preventing the PD-1 and PD-L1 interaction [122]. PCSK9 inhibition can also synergize with other strategies that increase the expression or function of TCR, such as interleukin-2 (IL-2), CD28 costimulation, or CAR-T-cell therapy [122,138]. Therefore, targeting PCSK9 represents a novel approach to modulate TCR expression and enhance the antitumor effectiveness of ICT.

### Stimulation of CD8^+^ T-cell infiltration and Treg cell exclusion

Another mechanism by which inhibiting PCSK9 boosts the antitumor efficacy of anti-PD-1/PD-L1 immunotherapy involves modulating the composition and function of TIIC [19,122]. TIICs consist of various cell types that can enhance or suppress tumor growth and immunity, depending on their type, ratio, and activation state [139]. Among TIICs, CD8^+^ T cells and Treg cells are 2 important subsets with opposite roles in tumor immunity. CD8^+^ T cells are cytotoxic lymphocytes that can directly eradicate tumor cells and generate proinflammatory cytokines, including IFN-γ and tumor TNF-α [140]. Treg cells are immunosuppressive lymphocytes capable of inhibiting the activation and function of CD8^+^ T cells and other effector cells by generating anti-inflammatory cytokines, including IL-10 and transforming growth factor-β (TGF-β) [141]. Therefore, the equilibrium between CD8^+^ T cells and Treg cells in the TME is vital for determining the outcome of tumor immunity and therapy.

PCSK9 inhibition can shift the balance of TIIC to favor CD8^+^ T cells and against Treg cells, thereby potentiating the antitumor effectiveness of anti-PD-1/PD-L1 immunotherapy [19]. Wang et al. demonstrated that inhibiting PCSK9 enhances the infiltration of CD8^+^ T cells while reducing the up-regulation of Treg cells in tumor models treated with an anti-PD-1 antibody [19]. PD-1 blockade increased the infiltration of Treg and CD8^+^ T cells within CT26, MC38, and CRC tumor models [19]. After that, they combined the anti-PD-1 therapy with an anti-PCSK9 antibody and detected PD-1 blockade-induced Treg cells and tumor-infiltrating CD8^+^ T cells [19]. The results indicated that targeting PCSK9 potentiated the infiltration of CD8^+^ T cells and eliminated the PD-1 blockade-induced increase in Treg cells. Furthermore, Yuan et al. proved that PCSK9 treatment could reduce the expression of the cytokines IFN-γ and TNF-α, cytokines that activate TIIC in the TME, in CD8^+^ T cells [122]. These results indicated that PCSK9 inhibition not only modulates the quantity but also improves the quality of TIIC in the TME, suggesting that PCSK9 inhibition influences the trafficking of TIIC and regulates the cytokine profile of the TME.

Inhibition of PCSK9 emerges as a compelling strategy to augment antitumor immunity, particularly through the enhancement of CD8^+^ T-cell infiltration and the concomitant reduction of Treg cell populations within the TME. This synergistic dual action is pivotal for shifting the equilibrium in favor of a potent antitumor immune response. The activation of CD8^+^ T cells, which are instrumental in the direct targeting and elimination of neoplastic cells, alongside the suppression of immunosuppressive Treg cells, fosters a more vigorous immune reaction against cancer [142]. The conception of innovative approaches that target immune checkpoints, such as PCSK9 inhibition, is underscored for its capacity to amplify the efficacy of immunotherapeutic interventions, notably those in conjunction with anti-PD-1/PD-L1 therapies [143]. Rastin et al. [144] discussed innovative delivery systems for immunotherapeutic agents, which could potentially be applied to PCSK9 inhibitors to ensure precise and targeted delivery to the tumor site. Mashhadi et al. [145] synthesized the existing knowledge regarding PCSK9’s implications in cancer prognosis and its emergent role as an immune therapy target. It suggests that inhibiting PCSK9 not only affects cholesterol metabolism but also modulates the immune response, making it a multifaceted target for cancer treatment [145]. Collectively, these insights suggest the capacity of PCSK9 inhibition to reconfigure the immunological milieu within the TME, thereby potentially enhancing the therapeutic efficacy of immunotherapies. Nonetheless, further in-depth research is imperative to elucidate the underlying mechanisms and to effectively translate these scientific discoveries into tangible clinical applications.

## Combined Applications of PCSK9 Inhibitors and Anti-PD-1/PD-L1 Therapy in the Clinic

Combining PCSK9 inhibition with anti-PD-1/PD-L1 immunotherapy has yielded promising preclinical results [146], which enhance the antitumor immune response and overcoming resistance to ICT. However, this combination strategy’s clinical evidence and applications are still limited and need to be further explored and validated. This section reviews the current clinical trials evaluating the efficacy and safety of PCSK9 inhibition and anti-PD-1/PD-L1 immunotherapy in different types of cancer and discusses the potential benefits and challenges of this novel therapeutic approach.

To date, 6 relevant clinical studies have been performed (ClinicalTrials.gov, Table 3). Among them, the first enrolled study (NCT03337698) was a phase Ib/II, open-label, multicenter, randomized umbrella study launched by Smilow Cancer Hospital at Yale New Haven and 37 additional locations; this study started on 2018 January 2. This study evaluated the safety, effectiveness, and pharmacokinetics of multiple immunotherapy combination therapies in individuals with metastatic non-small cell lung cancer (NSCLC). In this study, one of the treatment arms was a combination of atezolizumab, a PD-L1 inhibitor, and evolocumab, a PCSK9 inhibitor. The main endpoint was the percentage of patients with an objective response. In addition, the first relevant study enrolled in China (NCT05128539) was an open-label phase I clinical study launched by Henan Tumor Hospital. This study evaluated the safety, tolerability, pharmacokinetics, and initial efficacy of JS002 (Ongericimab), a bioengineered humanized anti-PCSK9 monoclonal antibody, combined with JS001 (Toripalimab), a PD-1 inhibitor, in individuals with progressive cancer for whom standard therapy has failed. The primary outcome measures were adverse event (AE), serious adverse event (SAE), immune-related adverse event (irAE), dose-limiting toxicity (DLT), maximum tolerated dose (MTD), and recommended dose for extension (RDE). Furthermore, the first enrolled randomized controlled trial (RCT) (NCT05144529) was a randomized pilot study launched by Duke University Medical Center. This study combined evolocumab (a PCSK9 inhibitor) with nivolumab (a PD-1 inhibitor) and ipilimumab (a cytotoxic T-lymphocyte-associated protein 4 [CTLA-4] inhibitor) in patients with metastatic NSCLC. This study aimed to determine the safety and tolerability of consuming evolocumab with standard immunotherapy in individuals with advanced lung cancer. The primary outcome measures were DLT and changes in CD3^+^ T cells. By combining a PCSK9 inhibitor with an immune checkpoint inhibitor, these trials hope to achieve synergistic effects of increasing tumor immunogenicity and overcoming PD-1/PD-L1-mediated immune suppression.

**Table 3.** Clinical trials of PCSK9 inhibitors combining with anti-PD-1/PD-L1 immunotherapy

Study title	ClinicalTrials.gov ID	Status	Locations	Actual/estimated study start date	Estimated enrollment	Conditions	Interventions	Study design
A study of multiple immunotherapy-based treatment combinations in participants with metastatic non-small cell lung cancer (Morpheus–non-small cell lung cancer)	NCT03337698	Recruiting	Smilow Cancer Hospital at Yale New Haven New Haven, Connecticut, USA (and 37 more)	2018 January 2	675	Carcinoma, NSCLC	Drug: Atezolizumab Drug: Cobimetinib Drug: RO6958688 (and 15 more)	Multi-arm trial
A study exploring JS001+JS002 in patients with advanced cancer	NCT05128539	Recruiting	Henan Tumor Hospital Zhengzhou, Henan, China	2021 December 23	114	Advanced cancer	Drug: JS001(Toripalimab) + JS002	Single-arm trial
A randomized pilot study of evolocumab plus nivolumab/ipilimumab in treatment-naïve patients with metastatic NSCLC	NCT05144529	Recruiting	Duke University Medical Center Durham, North Carolina, USA	2022 March 22	38	Lung cancer metastatic	Drug: Nivolumab Drug: Ipilimumab Drug: Evolocumab	Randomized controlled trial
PCSK9 inhibitor and PD-1 inhibitor in patients with metastatic, refractory to prior anti-PD1 non-small cell lung cancer	NCT05553834	Recruiting	Duke University Durham, North Carolina, USA	2023 May 16	60	NSCLC	Combination product: Alirocumab and Cemiplimab	Single-arm trial
A phase II study bolstering outcomes by optimizing immunotherapy strategies with evolocumab and nivolumab in patients with metastatic renal cell carcinoma (BOOST-RCC)	NCT06284564	Not yet recruiting	MD Anderson Cancer Center Houston, Texas, USA	2024 July 31	10	Metastatic renal cell carcinoma	Drug: Evolocumab Drug: Nivolumab	Single-arm trial
Neoadjuvant chemoradiotherapy combined with PD-1 inhibitor and PCSK9 inhibitor for pMMR/MSS locally advanced mid-low rectal cancer	NCT06304987	Not yet recruiting	Beijing Friendship Hospital, Capital Medical University Beijing, Beijing, China Beijing Friendship Hospital Beijing, Beijing, China	2024 April 1	50	Locally advanced rectal cancer	Combination product: Long-course chemoradiation and PD-1 inhibitor, with PCSK9 inhibitor	Randomized controlled trial

PCSK9, proprotein convertase subtilisin/kexin type 9; PD-1, programmed cell death protein 1; pMMR, mismatch repair-proficient; MSS, microsatellite stable; NSCLC, non-small cell lung cancer.

Our center also initiated a multicenter, prospective, randomized controlled study (NCT06304987) to evaluate the effectiveness and safety of neoadjuvant chemoradiotherapy combined with PD-1 blockade (Sintilimab) and a PCSK9 inhibitor (Tafolecimab) for treating pMMR/MSS locally advanced rectal cancer (LARC). Fifty LARC patients whose distance from the distal border of the tumor to the anal verge was ≤10 cm were consecutively enrolled and randomized (1:1) into the control or experimental group. Patients in the control group will receive long-term radiotherapy (50 Gy/25 fractions, 2 Gy/fraction, 5 days/week), three 21-day cycles of capecitabine (850 to 1,000 mg/m^2^, bid, po, days 1 to 14), and three 21-day cycles of PD-1 blockade (200 mg, iv. gtt, day 8) as neoadjuvant therapy. For the experimental group, patients will receive long-term radiotherapy (50 Gy/25 fractions, 2 Gy/fraction, 5 days/week), three 21-day cycles of capecitabine (850 to 1,000 mg/m^2^, bid, po, days 1 to 14), three 21-day cycles of PD-1 blockade (200 mg, iv. gtt, day 8), and a PCSK9 inhibitor (600 mg, ih, week 1 and week 7) twice as neoadjuvant therapy. The primary endpoint was the complete response (CR) rate, which was defined as the rate at which patients reached clinical complete response (cCR) or pathological complete response (pCR). The secondary endpoints were major pathological response rate (MPR), objective response rate (ORR), disease-free survival (DFS), overall survival (OS), organ preservation rate (OPR), neoadjuvant rectal cancer score (NAR), and quality of life score (QoL). We hope that this strategy will improve the organ preservation rate and quality of life of LARC patients without affecting survival outcomes, and we will further explore a pathway to enhance the effectiveness of ICT for treating pMMR/MSS patients.

## Summary and Outlook

This review provides an overview of the current insights into the role of PCSK9 in cholesterol metabolism and cancer biology and discuss the potential benefits and challenges of combining PCSK9 inhibition with anti-PD-1/PD-L1 immunotherapy for cancer treatment. PCSK9 is a serine proteinase that regulates cholesterol metabolism by attaching to LDLR and directing it for lysosomal degradation [99]. PCSK9 also interacts with other membrane receptors, such as MHC I, and affects their trafficking and stability [126]. PCSK9 inhibition can lower serum cholesterol levels and suppress tumor growth by modulating tumorigenic signaling pathways, ferroptosis, and the TME [117,118,120,122]. Moreover, PCSK9 inhibition can enhance the antitumor effectiveness of anti-PD-1/PD-L1 immunotherapy by restoring MHC I recycling and antigen presentation and promoting LDLR-mediated TCR recycling and signaling [122,126]. Therefore, targeting PCSK9 represents a novel approach to modulate cholesterol metabolism and enhance the antitumor effect of ICT.

However, some limitations and challenges need to be addressed before applying this combination strategy in clinical application. First, the optimal dose, timing, and duration of PCSK9 inhibition and anti-PD-1/PD-L1 immunotherapy need to be determined based on the tumor type, phase, and molecular characteristics. Second, the safety and tolerability of PCSK9 inhibition and anti-PD-1/PD-L1 immunotherapy need to be evaluated, especially in patients with cardiovascular diseases, diabetes, or other comorbidities. Third, biomarkers and predictors of response and resistance to PCSK9 inhibition and anti-PD-1/PD-L1 immunotherapy, such as PCSK9 expression, LDLR expression, MHC I expression, the TCR repertoire, and tumor mutational burden, need to be identified and validated. Fourth, the mechanisms of interaction and synergy between PCSK9 inhibition and anti-PD-1/PD-L1 immunotherapy, such as the effects of PCSK9 inhibition on other immune cells, cytokines, and chemokines in the TME, need to be further elucidated. Fifth, the prospect of combining PCSK9 inhibition and anti-PD-1/PD-L1 immunotherapy with other therapeutic modalities, such as chemotherapy, radiotherapy, targeted therapy, or other immunotherapies, needs to be explored and optimized. Therefore, the full realization of this combined therapeutic potential awaits further preclinical and clinical validation.

In conclusion, the convergence of PCSK9 inhibition with anti-PD-1/PD-L1 immunotherapy presents a multifaceted approach to cancer treatment, offering a tapestry of benefits that extend beyond the conventional therapeutic modalities. This synergistic combination not only modulates cholesterol metabolism but also invigorates the antitumor immune response, providing a double-edged sword against cancer. The distinct advantage of PCSK9 inhibitors lies in their non-cytotoxic nature, which results in a sensibly lower incidence of adverse reactions compared to traditional chemotherapeutic agents and radiation therapies. This lower toxicity profile contributes to an improved safety margin, making it an attractive option for patients who may not tolerate other aggressive treatments. Emerging evidence highlighting the link between tumors and hyperlipidemia further underscores the potential of PCSK9 inhibitors. This correlation suggests that the metabolic anomalies associated with hyperlipidemia might be harnessed to bolster the antitumor effects of immunotherapy, offering a dual therapeutic benefit. Particularly for obese patients, this combination could address both their metabolic and oncologic challenges simultaneously. The ease of administration of PCSK9 inhibitors, typically through subcutaneous injection, enhances patient compliance and simplifies treatment regimens. This convenience, coupled with the therapeutic effectiveness, may sensibly improve the quality of life for patients undergoing cancer treatment. In summary, the fusion of PCSK9 inhibition and anti-PD-1/PD-L1 immunotherapy heralds a new era in cancer therapeutics, with the promise of a safer, more patient-friendly, and potentially more effective treatment option. As we stand on the precipice of this novel therapeutic frontier, the scientific community eagerly anticipates the forthcoming research that will undoubtedly refine and expand our understanding, paving the way for improved cancer care.
